# Imageless ROSA® Robotic Arm-Assisted Total Knee Arthroplasty Is Associated With Improved Accuracy Compared to Conventional Techniques

**DOI:** 10.7759/cureus.96782

**Published:** 2025-11-13

**Authors:** Christopher L Hoehmann, Jeffrey Baker, Kyle Struck, Emily Fahey, Kristin Delfino, D Gordon Allan

**Affiliations:** 1 Department of Orthopedic Surgery, Southern Illinois University School of Medicine, Springfield, USA; 2 Center for Clinical Research, Southern Illinois University School of Medicine, Springfield, USA

**Keywords:** knee alignment, range of motion, robotic knee arthroplasty, rosa knee system, total knee arthroplasty

## Abstract

Background

The purpose of this study was to compare conventional and robotic-assisted total knee arthroplasty (TKA) using the imageless Robotic Surgical Assistant (ROSA®) Knee System.

Methods

This prospective study consecutively analyzed 23 patients undergoing conventional TKA against 27 patients undergoing robotic-assisted TKA and followed them for 20.8±2.0 and 17.6±1.3 months, respectively. All procedures were performed by a single surgeon, and radiographic measurements were taken by two observers.

Results

There were no major differences between the cohorts for age (p=0.40), sex (p=0.59), and body mass index (p=0.23). The groups were similar for length of stay (p=0.76), patellar resurfacing (p=0.27), cementation (p=0.23), and posterior cruciate ligament (PCL) resection (p=0.39). The robotic cohort more often used a thicker polyethylene bearing (p=0.01). Surgery was longer in the robotic cohort (78.3±2.0 versus 70.1±1.8; p=0.01). Differences were noted in radiographic alignment in coronal and sagittal planes, with the robotic cohort more frequently meeting criteria for functional alignment versus the conventional group (90.1% versus 34.9%, respectively; p=0.001). The intra-class correlation coefficient was excellent (0.75-1.00) for most recorded values. However, there did not appear to be meaningful differences between groups in terms of range of motion (ROM) and patient satisfaction.

Conclusions

The robotic cohort was found to be superior to the conventional cohort in terms of radiographic alignment in all planes, but similar in ROM and patient satisfaction.

## Introduction

Total knee arthroplasty (TKA) is commonly performed to treat knee arthritis [1]. Despite generally favorable results, approximately 10-20% of patients remain unsatisfied [1-4]. Although dissatisfaction is likely multifactorial, aberrations in knee alignment could be a contributing factor. A misaligned TKA, which is a TKA that is aligned aberrantly from what is considered functional or within what is the expected alignment of the limb, could become clinically relevant as it presents an unbalanced distribution of weight contributing to pain, stress, and implant failure [3,4].

Using conventional methods, there are approximately 10-20% of TKA alignment outliers greater than 3 degrees [2]. This has led to an interest in alternative alignment techniques, many of which include robotic-assisted surgery. A recent meta-analysis demonstrated that robotic-assisted TKA produced more accurate placement of components [5]. However, the systems investigated require pre-operative CT imaging, which is costly and exposes the patient to radiation [5,6]. Additionally, this study notably lacked data regarding the ROSA® (Robotic Surgical Assistant) Knee System (Zimmer Biomet, Montreal, Canada), which utilizes the most popular TKA implants worldwide [5,7,8]. In regards to the ROSA Knee, the literature is limited and mixed, with studies missing comparison groups, relying on cadaveric data, or being restricted to surgical technique descriptions [9-11].

There are several methods to compare TKA techniques. Differences in alignment are directly measurable; however, other outcomes are more difficult to elucidate. The literature is conflicted as to whether robotic-assisted TKAs are associated with better outcome measures and range of motion (ROM) [12-15]. Polyethylene thickness has been used as a measure for intra-operative accuracy; a large polyethylene component can result from inaccurate cuts and is a risk for implant failure [16].

In the present study, we aim to demonstrate the effectiveness of the ROSA Knee System when compared to conventional techniques in terms of radiographic alignment, polyethylene thickness, ROM, and patient satisfaction.

## Materials and methods

Study design

**Figure 1:**
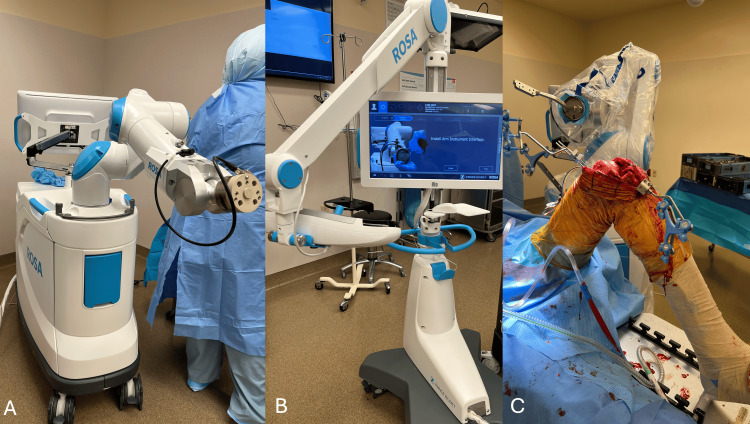
Clinical depiction of the robotic surgical assistant (ROSA Knee) from Zimmer Biomet. Panel A: Demonstrates the first mobile unit with a robotic arm. The cutting jig is attached sterilely to this arm. Panel B: The second mobile unit has an interactive screen where surgical planning can be performed and a sensor that monitors the movement of the trackers. Panel C: Demonstrates an intra-operative photo demonstrating trackers pinned into the femur and tibia of a knee, and the robotic arm with an attached jig that will be utilized to make bone resections.

Approval was obtained from the Institutional Review Board (#24-470). Fifty-eight patients with symptomatic knee osteoarthritis undergoing primary TKA between October 7, 2020, to June 25, 2021, were chosen for this study. Patients were included if they were between 50 and 90 years of age and had a primary diagnosis of osteoarthritis. Exclusion criteria included the following: any surgical indication other than primary knee osteoarthritis (fracture or previous osteotomy), conversion arthroplasty, or previous knee infection. All conventional surgeries were performed consecutively, followed by all robotic cases. This produced 27 patients in the control group and 27 patients in the robotic-assisted cohort. Four patients in the control cohort were excluded because of insurance denial. All surgeries were performed by a single fellowship-trained arthroplasty surgeon with more than 20 years of experience at three different institutions. This surgeon had been utilizing this implant system for five years and the ROSA Knee for nearly two years before the study; therefore, no patients were excluded for a learning curve period [17] (Figure 1). Funding was obtained from Zimmer Biomet as part of a larger multi-center prospective trial (NCT03969654).

Data collection

After the follow-up period, data were extracted from the patient charts, including demographics and physical exam findings. At each visit, the patients answered a health assessment as part of an EuroQol visual analogue scale (EQ-VAS) questionnaire, which was analyzed. As part of this questionnaire, patients reported their level of satisfaction on a scale between 0% and 100%. The operative report was further reviewed to note laterality, operative time, surgical technique, and polyethylene thickness. Radiographs were then measured by two independent arthroplasty-trained orthopaedic surgeons.

Radiographic measurements

**Figure 2:**
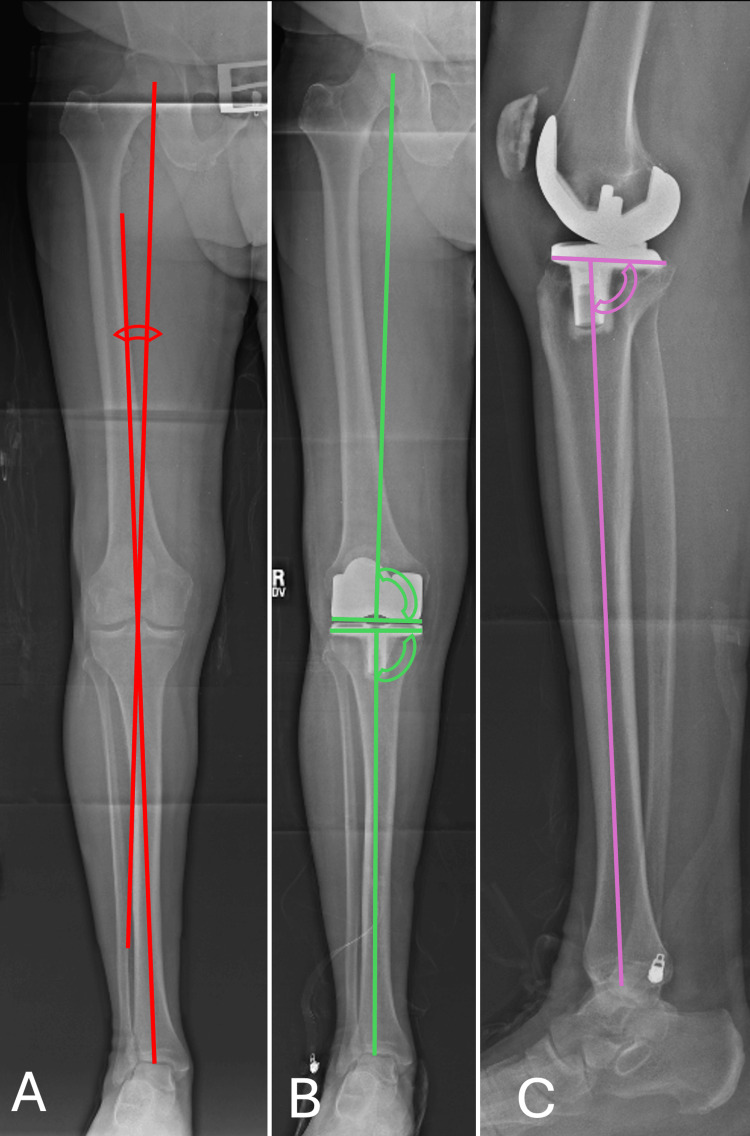
Measurements were recorded via plain radiographs. Panel A: The hip-knee-ankle angle (HKA) is measured in the right knee. Two lines are drawn—one from the center of the femoral head to the center of the intercondylar notch (representing the mechanical axis of the femur) and another from the center of the tibial plafond to the center of the tibial eminences (representing the mechanical axis of the tibia). The angle subtended by these two lines is the HKA. Panel B: The coronal inclination of the tibial and femoral components is measured as an angle between the mechanical axis of the named bone and the joint line of its prosthesis. Panel C: The sagittal inclination of the tibial component is measured using the long-leg lateral radiograph. An angle is measured between the long axis of the tibia and the tibial baseplate. The center of the ankle is used as a distal reference.

Standing AP and lateral long-leg films were utilized to measure alignment. Images were obtained preoperatively and six weeks postoperatively. Hip-knee-ankle alignment (HKA) was measured by determining the angle subtended between the mechanical axis of the femur and tibia. Femoral component positioning was measured by determining the angle subtended between the mechanical alignment of the femur and the joint line created by the femoral component. Tibial component positioning was determined by measuring the angle between the mechanical alignment of the tibia and the joint surface created by the tibial baseplate. Finally, the coronal inclination of the tibial component was measured as an angle formed between the mechanical axis of the tibia and the joint surface created by the tibial baseplate. These measurements (Figure 2) have been previously used to investigate limb alignment following TKA [13]. As has been described in the literature, we felt that functional alignment was achieved if the following criteria were met: femoral component within 6° of valgus and 1° of varus, tibial component within 5° of varus and 1° of valgus, and final HKA to be within 5° of varus and 3° of valgus [18]. These criteria of functional alignment are expected following total knee replacement when goals are consistent with standard mechanical means [18].

General surgical technique

A tourniquet was not applied. A midline skin incision was followed by a medial parapatellar approach. The anterior cruciate ligament was sacrificed, but the posterior cruciate ligament (PCL) was released at the surgeon’s discretion from intra-operative balancing. Osteophytes were removed before balancing. The retropatellar fat pad was retained. If patellar resurfacing was performed, it was completed before other osteotomies. All femoral resections were made before the tibial osteotomy. Osteotomies were planned based on the mechanical axis. The Persona® Knee System, designed by Zimmer Biomet (Warsaw, IN), was utilized in all cases with a medial congruent polyethylene insert. A decision for press fit versus cementation was made during the time of surgery while considering the patient’s bone quality and demographic factors. Recent data demonstrate similar survivorship between press-fit and cemented total knee replacements [19].

Conventional technique

Femoral resection was made from an intramedullary reference with a valgus angle of 4-7 degrees, which was determined using pre-operative imaging. Extramedullary tibial referencing was used to make a tibial osteotomy with a 3° posterior slope.

Robotic technique

The ROSA Knee was used as recommended by the manufacturer and as described in the literature [10]. The machine was calibrated via waypoint acquisition, marking femoral and tibial resections. ROM and balancing were performed before resections to assist with decision-making regarding implant position. This surgeon planned for flexion and extension gaps of 21 mm, as he felt this was the appropriate amount of tension and was consistent with the described technique [10]. After confirmation, the ROSA Knee machine self-positioned for resections; intraoperative adjustments and "re-cuts" were tracked as well. The trial implants were placed, and balancing was reassessed before final implantation.

Statistical analysis

Descriptive statistics were computed for all study variables. Categorical variables are described as frequencies and percentages. Comparisons between case and control groups were performed using Fisher’s exact test. Continuous variables are described with measures of central tendency (mean, median) and dispersion (range, standard deviation). Differences between case and control groups were tested with a t-test or the non-parametric equivalent, depending on the distribution. Paired t-tests were used to assess the pairwise comparisons of repeated measures.

A mixed model for repeated measures was used to examine the effect of group (case, control), time (baseline, post six weeks, post three months, post one year), and the interaction of group x time on the outcome measures ROM and health (EQ-VAS), separately. Models with a significant interaction effect were investigated further by testing the differences between the simple main effects. Models without a significant interaction, but with a significant main effect, were followed up with post-hoc tests of pairwise comparisons. P-values were adjusted for multiple testing with a Bonferroni correction.

An overall intraclass correlation statistic was computed for the two raters based on all of their continuous variable measures. Inter-rater reliability for the measured categorical data of the two raters was assessed with Cohen’s kappa. Intraclass correlation coefficient values were classified as follows: excellent=0.75-1.00, good=0.60-0.74, fair=0.40-0.59, and poor=less than 0.40 (15). P-values less than 0.05 were considered statistically significant. All data were analyzed with SAS v9.4.

## Results

A total of 50 patients were included for analysis. Mean age at surgery was 69.6 ± 7.6 years, mean BMI was 31.7 ± 5.1, 52% were male, and 98% were Caucasian. Table 1 demonstrates patient sample characteristics for case and control patients. The groups were similar in terms of age (p=0.40), sex (p=0.59), race, body mass index (p=0.23), and medical and surgical history.

The mean age was near 70 years (range: 53-88), 56.5% of the control cohort, 48.1% of the case cohort were men, BMI was slightly above 30 in both groups, and both groups had a single patient with a previous ipsilateral knee arthroscopy. There was no significant difference in time to discharge between the control and case cohorts (9.2 (8.1-10.0) and 9.3 (8.0-13.2), respectively). Two of the control cases occurred in an ambulatory surgical center, whereas all other cases occurred at two different hospitals (Table 1).

**Table 1:** Comparison of demographics, medical risk factors, and surgical variables between cases and control groups. SD, standard deviation; BMI, body mass index; IQR, Interquartile Range; PCL, posterior cruciate ligament P-values calculated using Fisher’s exact test for categorical variables with small expected counts; no test statistic is reported for these comparisons.

Characteristics	Control	Case	P value	Test Statistic	Test Statistic Reported	Test Used
	n=23	n=27				
Age, years, mean ± SD	68.6 ± 8.2	70.5 ± 7.0	0.379	-0.890	t value	T-test
Sex, n (%)			0.584			Fisher's exact
Male	13 (56.5)	13 (48.1)				
Female	10 (43.5)	14 (51.9)				
Race, n (%)						
White	23 (100.0)	26 (96.3)				
Black	0 (0.0)	0 (0.0)				
Hispanic/Latino	0 (0.0)	0 (0.0)				
Asian	0 (0.0)	1 (3.7)				
BMI, mean ± SD	32.6 ± 5.5	30.9 ± 4.8	0.233	1.210	t value	T-test
Diabetes Mellitus, n (%)	3 (13.0)	3 (11.1)	0.999			Fisher's exact
Rheumatoid Arthritis, n (%)	1 (4.4)	0 (0.0)				
Osteoporosis, n (%)	0 (0.0)	1 (3.7)				
Nicotine Dependence, n (%)	0 (0.0)	0 (0.0)				
Previous Knee Surgery, n (%)	1 (4.35)	1 (3.7)				.
Laterality (Right), n (%)	14 (60.9)	19 (70.4)	0.557			Fisher's exact
Operative time, mean ± SD	70.1 ± 8.5	78.3 ± 10.1	0.004	-3.070	tvalue	T-test
Length of stay (hours), median (IQR)	9.2 (8.1-10.0)	9.3 (8.0-13.2)	0.778	-0.282	Z	Mann Whitney
Length of stay (days), median (IQR)	0.4 (0.3-0.4)	0.4 (0.3-0.5)	0.778	-0.282	Z	Mann Whitney
Location, n (%)						
Hospital 1	14 (60.9)	13 (48.2)				
Hospital 2	7 (30.4)	14 (51.8)				
Ambulatory surgery center	2 (8.7)	0 (0.0)				
Polyethylene thickness, n (%)			0.040			Fisher's exact
10	20 (87.0)	14 (51.9)				
11	1 (4.4)	5 (18.5)				
12	2 (8.7)	4 (14.8)				
13	0 (0.0)	4 (14.8)				
Polyethylene above 10mm, n (%)	3 (13.0)	13 (48.2)	0.014			Fisher's Exact
Cemented Components, n (%)	18 (78.3)	25 (92.6)	0.225			Fisher's Exact
Patella Resurfaced, n (%)	17 (73.9)	24 (88.9)	0.270			Fisher's Exact
PCL Resected, n (%)	12 (52.2)	10 (37.0)	0.393			Fisher's Exact
Trays, n (%						
3	2 (8.7)	0 (0.0)				
4	0 (0.0)	1 (3.7)				
5	19 (82.6)	25 (92.6)				
6	1 (4.4)	1 (3.7)				
7	1 (4.4)	0 (0.0)				
Abnormal patellar tracking, n (%)	1 (4.4)	0 (0.0)				
Manipulations, n (%)	0 (0.0)	0 (0.0)				
Months between surgery and final, median (IQR)	19 (12.4-27.9)	15.1 (12.0-22.6)	0.200	1.283	Z	Mann Whitney

Operative descriptives were similar between the two cohorts. The operative time was slightly longer in the case cohort (78.3 ± 10.1 minutes versus 70.1 ± 8.5). In both the control and case cohorts, the right limb was operated on more frequently than the left (60.9% and 70.4%, respectively). A polyethylene bearing thickness greater than baseline was used more frequently in the case cohort: 48.2% (13 patients) versus 13% (three patients). This implant’s baseline polyethylene thickness is 10 mm, with a maximum of 20 mm. The frequency of re-cuts was not tracked in either cohort. There was one case of patellar maltracking in the control cohort and none in the case cohort. No manipulations were performed during the study; however, one patient in the robotic group underwent arthroscopic lysis of adhesions during the follow-up period. The groups were similar in terms of the frequency of patellar resurfacing (p=0.27), cementation (p=0.23), and PCL resection (p=0.39). There were no revision surgeries. Total follow-up time was similar, with a median (IQR) of 19 (12.4-27.9) months in the control group and 15.1 (12.0-22.6) months in the other (Table 1).

Table 2 compares the ROM between the cohorts. A total of 39% of controls (n=9) and 52% of cases (n=15) had no flexion contracture before or following surgery. Prior to surgery, approximately half of the patients presented with a flexion contracture (56.5% of controls versus 44.4% of cases). Postoperatively, flexion contracture persisted in 9% of controls and 15% of cases. Improvement from preoperative flexion contracture to no contracture postoperatively was 48% among controls compared to 30% of cases. Only one case patient (4%) developed a new flexion contracture following surgery, and one control patient (4%) had missing postoperative data.

**Table 2:** Comparison of the range of motion and self-reported health assessments between cases and control groups. SD, standard deviation; IQR, interquartile range

Characteristics		Control	Case	P value	Test Statistic	Test Statistic Reported	Test Used
		n=23	n=27				
Pre-Flexion Contracture, n (%)							
No Contracture		10 (43.5)	15 (55.6)				
1-5 Degrees		11 (47.8)	11 (40.7)				
6-10 Degrees		1 (4.4)	0 (0.0)				
11-15 Degrees		1 (4.4)	1 (3.7)				
Pre Flexion Contracture, n (%)		13 (56.5)	12 (44.44)	0.571			Fisher's exact
Pre Extensor Lag, n (%)		0 (0.0)	0 (0.0)				
Pre ROM, mean ± SD		111.7 ± 12.2	112.2 ± 16.1	0.718	-0.3615	Z	Mann Whitney
Post ROM 6 weeks, mean ± SD		108.3 ± 9.1	107.6 ± 13.7				
Post ROM 3 months, mean ± SD		115.7 ± 6.3	113.1 ± 11.0				
Post ROM 1 year, mean ± SD		117.3 ± 6.7	117.4 ± 8.6				
Post ROM Final – Flexion, mean ± SD		118 ± 6.1	118.9 ± 8.8	0.699	-0.3872	Z	Mann Whitney
Post ROM Final – Extension, mean ± SD		0.2 ± 1.1	0.4 ± 1.3	0.409	-0.8264	Z	Mann Whitney
Post Flexion Contracture, n (%)		2 (8.7)	5 (18.5)				
Post Extensor Lag, n (%)		0 (0.0)	0 (0.0)				

**Figure 3:**
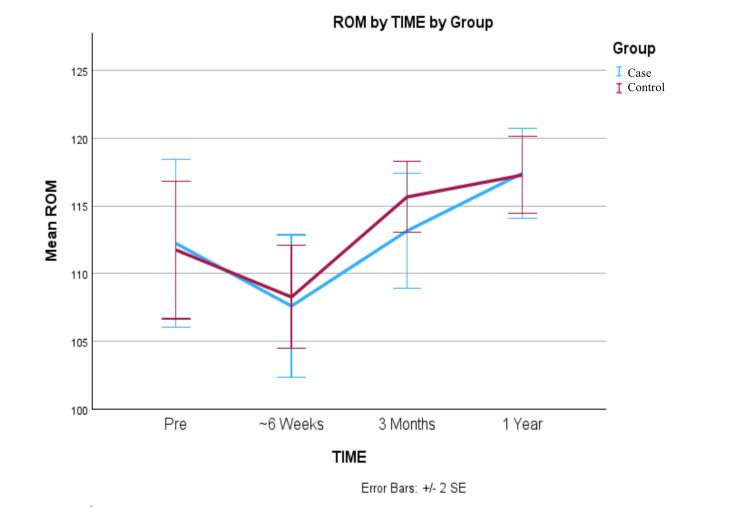
Line graph demonstrating the progression of the knee range of motion during the study period. The case group is resented in cyan blue whereas the controls are highlighted in magenta.

No patients had an extensor lag before or after surgery. There was no statistical difference in the ROM between either cohort at any time point (Table 2, Figure 3).

**Figure 4:**
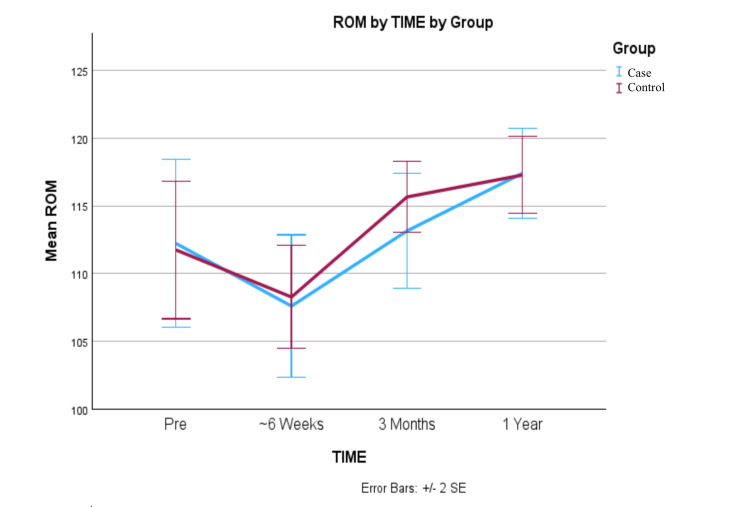
Line graph demonstrating the progression of a self-reported health assessment as part of the EQ-VAS self-assessment during the study period. The case group is represented in cyan blue, whereas the controls are highlighted in magenta.

Table 3 reports on the differences in self-reported health assessments as part of the EQ-VAS questionnaire. The majority of patients felt “very satisfied” after the study (n=15, 65% in the control group and n=20, 74.1% in the case group). Although a higher percentage of patients reported being “very satisfied” in the case group, there was a single patient who identified as “unsatisfied”; no patients in the control cohort reported this way. The difference in self-reported health between groups is consistent across all time points (Figure 4). The control group only reports on 22 patients, as one was not available to record a final satisfaction score.

**Table 3:** Comparison of the self-reported EQ-5D-5L VAS between cases and control groups. SD, standard deviation

Characteristics	Control	Case	P value	Test Statistic	Test Statistic Reported	Test Used
	n=23	n=27				
Pre Health, mean ± SD	79.3 ± 2.8	73.6 ± 2.7	0.152	1.460	t value	T-test
Post Health 6 weeks, mean ± SD	83.0 ± 2.3	79.1 ± 2.5				
Post Health 3 months, mean ± SD	83.5 ± 2.1	73.9 ± 2.7				
Post Health 1 year, mean ± SD	84.8 ± 2.4	77.0 ± 3.4				
Final Satisfaction, n (%)			0.938			Fisher's Exact
Very Satisfied	15 (65.2)	20 (74.1)				
Satisfied	5 (21.7)	4 (14.8)				
Uncertain	2 (8.7)	2 (7.4)				
Unsatisfied	0 (0.0)	1 (3.7)				

Table 4 describes knee alignment measurements. Both raters found significant differences in alignment between the cases and cohorts in terms of pre-operative alignment, post-operative alignment, and sagittal component alignment. Rater 1 found a difference between the groups in terms of tibial alignment; however, rater 2 did not. The case cohort more frequently met the criteria for functional alignment (n=26, 96.3%, in rater 1; n=23, 85.2% in rater 2) versus the control cohort, which often did not meet such criteria (n=6, 26.1%, in rater 1; n=10, 43.5%, in rater 2). The intraclass correlation coefficient between both observers was excellent (0.75-1.00) for all recorded values, except the tibial component was rated as good (0.60-0.74, 15; Table 4).

**Table 4:** Comparison of radiographic measurements between cases and control groups with intraclass correlation coefficients. SD, standard deviation; ICC, intra-class correlation; CI, confidence interval

Characteristics	Control	Case	P value	ICC (95% CI)	Test Statistic	Test Statistic Reported	Test Used
	n=23	n=27					
Pre Limb Alignment, mean ± SD	8.9 ± 4.7	4.0 ± 5.9	0.010	0.998 (0.997, 0.999)	2.5794	Z	Mann Whitney
Rater 1	8.9 ± 4.6	4.0 ± 5.9	0.013		2.4822	Z	Mann Whitney
Rater 2	9.0 ± 4.8	4.0 ± 5.9	0.007		2.7161	Z	Mann Whitney
Post Limb Alignment, mean ± SD	3.7 ± 2.4	1.9 ± 2.3		0.962 (0.927, 0.979)			
Rater 1	3.4 ± 2.5	1.9 ± 2.1					
Rater 2	4.0 ± 2.4	2.0 ± 2.6					
Change (Post-Pre) in Limb Alignment, mean ± SD	-5.2 ± 5.0	-2.1 ± 5.0	0.031		-2.22	t value	T-test
Rater 1	-5.5 ± 5.0	-2.2 ± 5.2	0.029		-2.25	t value	T-test
Rater 2	-5.0 ± 4.9	-2.0 ± 4.9	0.036		-2.16	t value	T-test
Femoral Component Alignment, mean ± SD	88.3 ± 2.5	88.9 ± 1.2	0.726	0.857 (0.750, 0.919)	-0.351	Z	Mann Whitney
Rater 1	88.1 ± 3.1	88.9 ± 1.2	0.350		-0.935	Z	Mann Whitney
Rater 2	88.4 ± 2.2	89.0 ± 1.3	0.838		-0.205	Z	Mann Whitney
Tibial Component Alignment, mean ± SD	90.2 ± 1.3	89.6 ± 0.9	0.090	0.632 (0.352, 0.791)	1.730	t value	T-test
Rater 1	90.5 ± 1.5	89.5 ± 0.9	0.007		2.680	Z	Mann Whitney
Rater 2	89.8 ± 1.4	89.8 ± 1.3	0.959		0.050	t value	Mann Whitney
Sagittal Component Alignment, mean ± SD	88.1 ± 2.7	85.1 ± 1.1	< 0.0001	0.866 (0.764, 0.924)	4.119	Z	Mann Whitney
Rater 1	88.3 ± 2.4	85.2 ± 0.9	< 0.0001		4.636	Z	Mann Whitney
Rater 2	88.0 ± 3.4	85.0 ± 1.6	0.001		3.495	Z	Mann Whitney
Functional Alignment, n (%)							There is no 'overall' analysis as raters did not meet and decide on one outcome, and is not continuous to allow an average.
Rater 1	6 (26.1)	26 (96.3)	< 0.0001				Fisher's Exact
Rater 2	10.0 (43.5)	23 (85.2)	0.0028				Fisher's Exact

## Discussion

We found that radiographic alignment was more accurate when using the ROSA Knee System but did not find any meaningful differences in terms of ROM or self-reported health assessments.

The case and control cohorts are fairly homogeneous and comparable. The robotic case cohort had slightly longer operative times than the control cohort, 78.3±2.0 versus 70.1±1.8 minutes, respectively. We attribute this to robotic setup and waypoint registration, which is required for surgical planning. Surgical factors remained uniform as well: the groups were similar in terms of utilizing cemented components, patellar resurfacing, number of trays opened, surgical location, and selective PCL resection. Though not statistically significant, a greater frequency of patients in the robotic group (about n=25, 93%, vs. n=18, 78%) received cemented implants, which could contribute to this time difference. The lack of significance could be related to the small sample size.

The groups were different in that a polyethylene insert above the planned baseline thickness was used more commonly in the robotic case cohort. As a thicker polyethylene is often used to compensate for increased bone loss, a thicker insert in the primary setting may mean that a larger-than-expected resection was performed [20]. This may be interpreted as the surgeon determined intra-operatively that the gaps made with the robotic arm were more often larger than expected and could question the accuracy of the cuts, planning, or the surgeon’s judgment. However, the thickest insert used was only 3 mm more than the baseline, and, therefore, we are unsure how to interpret this small difference clinically. Furthermore, the surgeon aimed for gaps of 21 mm, which is consistent with the described technique despite the implant thickness being 19 mm [10]. Perhaps, a goal of 21 mm is too large, and smaller gaps should be aimed for.

This study agrees with the existing literature that robotic technology provides more accurate TKA alignment [5-7,20-24]. However, this study supplements the paucity of literature surrounding the less explored ROSA Knee System. Imageless robotic technology has been shown to produce accurate alignment in cadavers but lacks support in human studies [9,25]. Shin et al. is one of the few reports to investigate these alignment aims with the ROSA Knee, which concluded that coronal alignment was accurate, but sagittal alignment was less accurate [7]. Our study was able to replicate their accuracy in the coronal plane, but we did not find the inaccuracies in the sagittal plane that they had encountered. Sagittal alignment is notoriously difficult to measure, which may be a reason for this discrepancy, in addition to their study’s lack of a comparison group [7,22]. Furthermore, our data demonstrate that the ROSA Knee was better able to produce criteria that met functional alignment than conventional techniques [7,18]. The intraclass correlation coefficients were excellent for almost all recorded values, and, therefore, we believe that the measurements obtained in this study can be considered reliable and fit for analysis [15].

Some studies have suggested that robotic-assisted TKA is associated with improved early recovery and ROM [14]. A prospective study by Kayani et al. showed that robotic-assisted TKA patients were discharged earlier and had better ROM [14]. However, their patients were discharged a mean of 77 and 105 hours after surgery for each cohort [14]. We feel that our study did not replicate these results because we performed mostly outpatient surgery [14]. Furthermore, this study only followed patients during their inpatient stay [14]. Our study, which did not find any meaningful differences in terms of ROM, followed patients for over a year. This could be extrapolated to mean that robotic TKA improves immediate ROM, but this difference is not sustained over time.

The effect of robotic assistance on functional outcomes and patient satisfaction has been a debated topic. A randomized control trial by Kim et al. [28] that followed 1,406 patients for 10 years did not demonstrate any difference in Knee Society, WOMAC, or UCLA activity scores [26]. Furthermore, a systematic review by Hoveidaei failed to demonstrate a difference in patient satisfaction scores [27]. Similarly, we found no meaningful differences in terms of a self-reported health assessment. We did, however, find that most patients identified as “very satisfied” after the study, 15 (65.2%) and 20 (74.1%) for the control and case cohorts, respectively, but there was not a noticeable difference between the groups. Conversely, a prospective study of 223 patients by Smith et al. demonstrated higher Knee Society Scores in the robotic group along with higher patient satisfaction scores [28-30]. The literature remains mixed on this topic.

There are some limitations of this study. The sample size is limited; however, a smaller group of patients allowed us to follow patients more closely and for a longer duration. All cases were performed by a single surgeon, which does introduce bias in terms of that surgeon’s skill and decision-making, but it also eliminates bias and confounding factors introduced by including multiple surgeons. Another limitation is that measurements relied on two-dimensional radiographs instead of three-dimensional cross-sectional imaging; however, we did include orthogonal imaging for analysis, which may help reduce this limitation. Although radiographs are technique and reader-dependent, we feel that this limitation was mitigated for several reasons: this imaging protocol was already established in this surgeon’s practice, and the technicians were proficient at this technique. Additionally, ICC demonstrated reproducible measurements of these radiographs between independent raters. Most other studies that explore TKA alignment also rely on radiographic imaging. It should be noted that this study does not attempt to establish a link between long-term outcomes associated with alignment following total knee arthroplasty.

A strength of this study was that the surgeon had significant experience with conventional techniques and also the ROSA Knee, which eliminates any learning curve that may be present in other studies. Furthermore, the consecutive design allowed a head-to-head comparison between the treatment arms.

## Conclusions

This study demonstrates the effectiveness of the imageless ROSA Knee System when compared to conventional techniques. The robotic system was found to be superior in terms of radiographic alignment. However, the ROSA cohort was comparable to the conventional cohort in terms of patient satisfaction and range of motion. The ROSA Knee System seems to demonstrate improved surgical accuracy, but this study did not demonstrate a meaningful clinical difference between the cohorts.
